# Atypical manifestation of giant thyroid goiter: a case report presenting with Arm paresthesia

**DOI:** 10.3389/fsurg.2025.1619195

**Published:** 2025-08-15

**Authors:** Po-Yu Chiu, Che-Hsuan Lin, Shih-Chun Lu, Hann-Ziong Yueh

**Affiliations:** ^1^ Department of Otolaryngology, Taipei Medical University Hospital, Taipei Medical University, Taipei, Taiwan; ^2^ Department of Otolaryngology, School of Medicine, College of Medicine, Taipei Medical University, Taipei, Taiwan

**Keywords:** goiter, benign, thyroid nodule, brachial plexus, case report

## Abstract

A 61-year-old male presented with persistent numbness, weakness, and soreness in his left arm. Imaging and fine-needle aspiration cytology indicated a benign thyroid nodule, which was subsequently confirmed by postoperative pathology. The patient recovered uneventfully following surgery, with no evidence of recurrence during follow-up. While large thyroid nodules typically cause tracheal compression, dysphagia, or compromised vascular flow, this rare case involved a mass effect compressing the brachial plexus, resulting in neurological symptoms such as numbness, weakness, and soreness.

## 1 Introduction

Thyroid nodules refer to abnormal growths of the thyroid gland arising from various etiologies, which may present as diffuse or nodular enlargement. They can be classified as non-toxic (euthyroid), toxic (thyrotoxic), or hypoactive (hypothyroid), depending on their functional status. Thyroid nodules may exert compressive effects on adjacent structures such as the trachea, recurrent laryngeal nerve, and esophagus, leading to clinical manifestations such as dyspnea or dysphagia. Typically, they present as painless neck masses. Globally, iodine deficiency remains the most common cause of thyroid nodules, whereas in iodine-sufficient regions, chronic autoimmune thyroiditis (Hashimoto's thyroiditis) and Graves' disease are more prevalent causes. In elderly populations, multinodular goiter is the most common etiology (1). Surgery or radioactive iodine therapy is the mainstay of treatment for large thyroid nodules with compressive symptoms (2). In this case, we highlight the presentation, diagnosis, and treatment of a middle-aged male patient with a giant nodular goiter accompanied by the mass effect.

## 2 Case presentation

**Figure 1:**
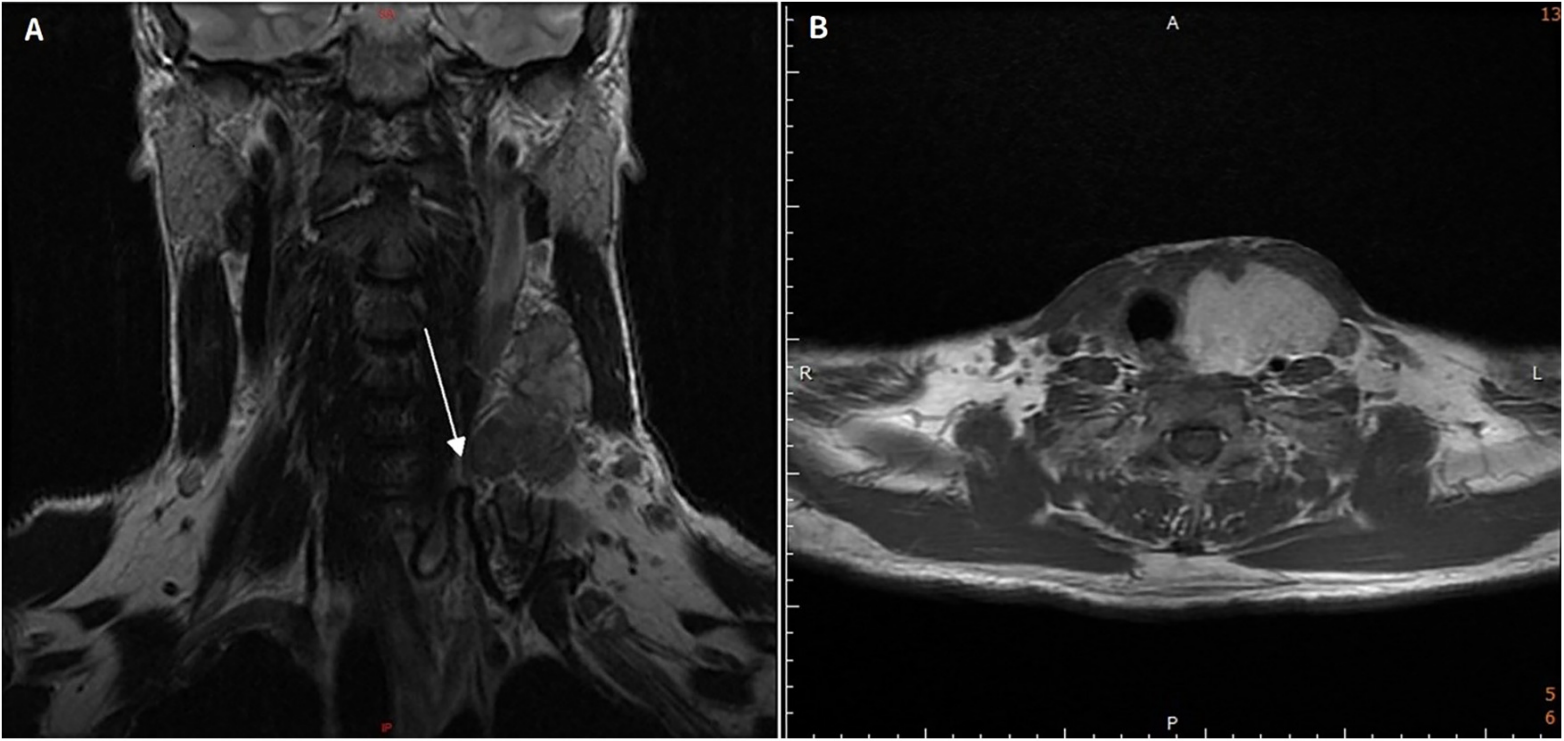
Pre-operative clinical and magnetic resonance images. **(A,B)** Showed a large cystic lesion with well-defined margins involving left thyroid gland, results in right deviation of the trachea.

A 61-year-old male patient initially presented to the cardiology department with hypertension and a gradually enlarging neck mass over the past few months. He subsequently developed left arm soreness, numbness, weakness, and tinnitus, prompting further evaluation at the otolaryngology outpatient department. His medical history was significant for essential hypertension and hyperlipidemia, both well-controlled with medication, and there was no family history of malignancies. Clinical assessment and laboratory investigations did not reveal any evidence of hyperthyroidism. Additionally, there were no associated symptoms of dysphagia, dyspnea, or voice changes. Physical examination revealed a large, round-shaped, soft, non-tender, well-defined mass in the left neck level III district, with no infiltration into surrounding soft tissues. No clinical signs indicative of Horner's syndrome such as ptosis, miosis, anhidrosis were observed. Fine-needle aspiration cytology (FNAC) revealed some hemosiderin-laden macrophages, suspected Inflammation. The patient also visited the neurology outpatient clinic, where the neurologist arranged a nerve conduction velocity (NCV) study for further evaluation. The NCV study is essentially normal (Supplementary Figure 2). Head and neck magnetic resonance imaging (MRI) showed a cystic lesion with well-defined margins, measuring 5.9 cm × 3.9 cm × 9.1 cm. The arteries and veins in the neck are displaced posteriorly and laterally due to the enlarged thyroid gland. The trachea appears compressed by the enlarged gland, and no retrosternal extension is observed (Figure 1).

**Figure 2:**
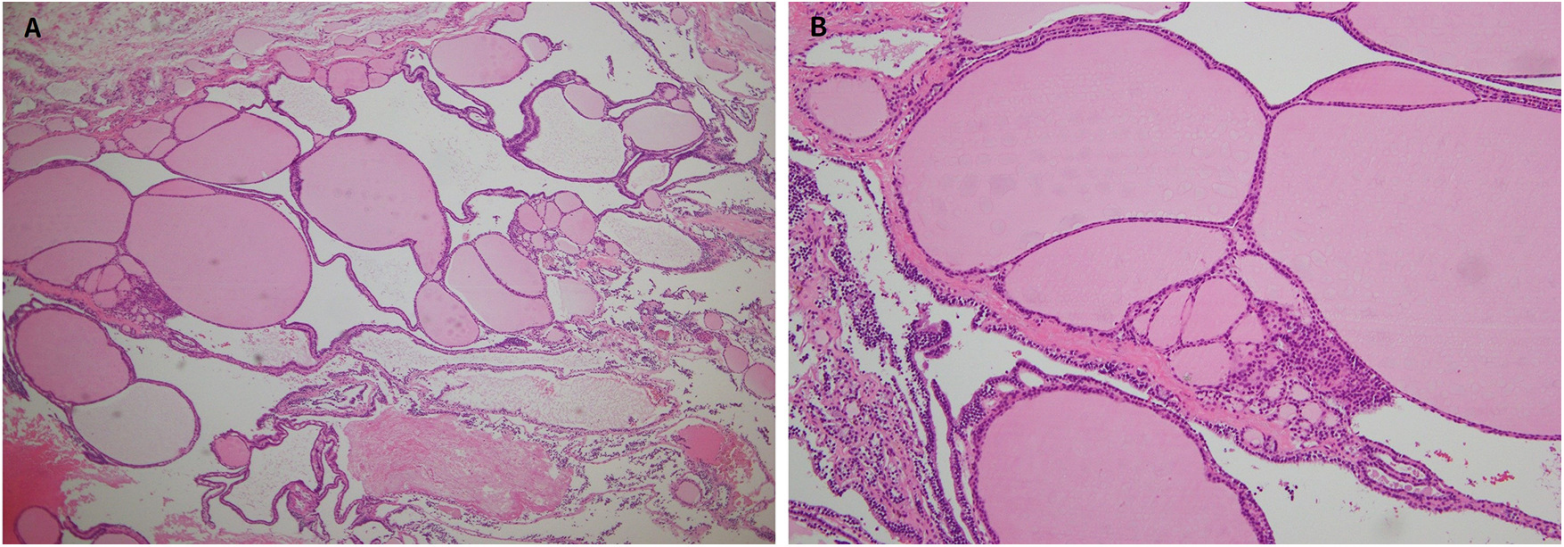
Histopathology of the thyroid gland under hematoxylin and eosin stain **(A)** 40×, **(B)** 100× showing variably sized follicles with flattened hyperplastic epithelium and fibrosis.

Imaging demonstrated that the tumor had well-defined borders, and FNAC primarily identified inflammatory cells, suggesting a higher likelihood of benignity. Furthermore, no common signs of malignancy, such as hoarseness or dysphagia secondary to nerve invasion, were observed. However, due to the patient's persistent complaints of numbness and aching in the left hand and shoulder, surgical excision of the mass was deemed appropriate. The patient underwent hemithyroidectomy as a treatment consideration. Hemithyroidectomy was performed with intraoperative neuromonitoring (IONM) to identify and preserve the recurrent laryngeal nerve. A standard system of Medtronic NIM was utilized with surface EMG recordings obtained via endotracheal tube electrodes. Stimulation was applied using a monopolar probe at an intensity of 1.0–2.0 mA, and signals exceeding 100 µV were considered valid responses. No specific neuromonitoring was performed for the brachial plexus, as the surgical field did not involve direct dissection surrounding its anatomical course. The improvement of neurological symptoms postoperatively supports the hypothesis of preoperative nerve compression rather than intraoperative injury. Pathological analysis indicated nodular hyperplasia of the thyroid, confirming a diagnosis of thyroid goiter (Figure 2). The drainage tube was removed on the fifth postoperative day, and the patient was discharged without complications. Postoperatively, B-complex vitamins and analgesics were prescribed. At the postoperative day 5 follow-up visit, the patient reported complete resolution of symptoms including arm paresthesia and muscle weakness. Subsequent outpatient follow-ups, including sonographic examinations, showed no evidence of tumor recurrence.

## 3 Discussion

Non-toxic goiter, also known as simple goiter, often occurs regionally and is commonly related to dietary habits. It typically does not present with symptoms of hyperthyroidism or hypothyroidism. Common causes include iodine deficiency, goitrogenic substances, excessive iodine intake, and a lack of trace elements such as zinc and selenium. Simple goiter generally does not require surgery (1). Treatment mainly focuses on addressing the underlying causes unless there are symptoms of compression of adjacent tissues and organs, complications, or malignant transformation, which may then necessitate surgical intervention.

In general cases of large goiters, compression complications such as difficulty swallowing, breathing difficulties, and impaired blood return are relatively common (3–5). Only a very few cases involve the brachial plexus, and these are typically malignant tumors (6, 7). In this particular patient, swallowing and breathing functions were normal, and only the brachial plexus function was affected. The pathology report indicated a benign condition, highlighting the rarity of this case.

In the differential diagnosis of benign tumors in the anterior triangle of the neck, thyroid nodules are the primary consideration. Although cystic hygromas and lipomas typically arise in the posterior triangle of the neck, they can occasionally present in the anterior triangle and should be ruled out in such cases. Second branchial cleft cysts, located anterior to the sternocleidomastoid, must also be considered. Thyroglossal duct cysts, which typically present along the midline of the neck, are inconsistent with this patient's left-sided neck mass. Additionally, tuberculous lymphadenitis, which predominantly affects young women aged 20–40 years (8), is less likely in this case due to the patient's profile as a 61-year-old male.

The mechanism by which a large tumor volume compresses nerves and causes sensory and motor dysfunction primarily involves direct compression of the surrounding nerves by the tumor. This compression blocks the transmission of nerve signals, leading to sensory and motor function impairment. These symptoms may gradually worsen, and as the compression persists, the nerve damage may become irreversible. Common causes of nerve compression by tumors include the increase in the tumor's volume, local inflammation and swelling caused by the tumor, and vascular obstruction induced by the tumor, which leads to insufficient nutrient supply to the nerves, further exacerbating nerve damage (9). The brachial plexus originates from the spinal nerve roots C5 to T1 and is a triangular structure extending from the spinal cord to the axilla. It is further divided into three trunks, six divisions, three cords, and terminal nerves, responsible for conveying motor, sensory, and sympathetic innervation to the upper limb (10).

Imaging of the patient suggests that the tumor may be compressing the C3 to C7 regions, affecting the area from the left shoulder to the left forearm. Motor function in muscles such as the coracobrachialis, brachialis, biceps, triceps, brachioradialis, and extensor carpi radialis may be impacted. In terms of sensation, dermatomes from C3 to C7 may exhibit abnormal perception, including pain, numbness, tingling, or loss of sensation. Regarding autonomic function, the hypothalamus sends descending autonomic fibers to the spinal cord, where sympathetic preganglionic neurons originate from the intermediolateral cell column of T1 to L2. Sympathetic preganglionic neurons may exit at the T1 level, and enter the cervical sympathetic chain. Then nerve fibers ascend to the superior cervical ganglion at the C3–C4 level. When a mass compresses the spinal nerves in that region, the patient may result in autonomic dysfunction such as Horner's syndrome.

Generally, it is believed that benign non-toxic thyroid nodules diagnosed by FNA do not require surgery and can be managed by addressing the underlying condition, but there are some exceptions. Firstly, the presence or absence of symptoms is a consideration. There is a significant correlation between the size of thyroid nodules and compression of the trachea and blood vessels. Eng et al. (11) have shown that the average size of nodules in patients with compression symptoms is 3.8 cm. The most common symptom is dysphagia, occurring in 80% of patients, followed by neck fullness (69%), choking (49%), and dyspnea (32%). Binar et al. (12) indicates that when the thyroid volume reaches 19.75 ml, it causes a 10% narrowing of the trachea, and when the volume reaches 30.29 ml, it causes a 40% narrowing of the trachea. Eng et al. (11) also shows that patients with enlarged nodules on the left side are more likely to experience dysphagia compared to those with right-sided nodules; however, these differences are not statistically significant. Among symptomatic patients, 92.7% showed significant improvement after surgery. Regarding the risk of cancer in the context of positive FNA results, Bernet et al. (13) mentions that for nodules larger than 4 cm, the accuracy of Fine-needle aspiration biopsy (FNAB) ranges from 80.3% to 87.5%. McCoy et al. (14) notes that thyroid nodules larger than or equal to 4 cm have a higher malignancy rate (26%) and that FNAB has a 13% false-negative rate and a 34% rate of missing follicular lesions. It is recommended that a benign FNAB result should not change the decision to perform surgery on thyroid nodules larger than or equal to 4 cm.

## 4 Conclusion

A large thyroid gland can present with a variety of symptoms, the most common of which include difficulty swallowing, difficulty breathing, and hoarseness. In our case, the patient suffered from numbness, soreness and weakness in the upper limbs since the brachial plexus was compressed by the huge thyroid gland, which is a rare presentation and hardly reported in past literature. Surgical removal of the thyroid entirely is a proper and effective treatment.

## Data Availability

The original contributions presented in the study are included in the article/Supplementary Material, further inquiries can be directed to the corresponding author.
